# Vehicle Mode and Driving Activity Detection Based on Analyzing Sensor Data of Smartphones

**DOI:** 10.3390/s18041036

**Published:** 2018-03-29

**Authors:** Dang-Nhac Lu, Duc-Nhan Nguyen, Thi-Hau Nguyen, Ha-Nam Nguyen

**Affiliations:** 1 University of Engineering and Technology, Vietnam National University in Hanoi (VNU-UET), Hanoi 123105, Vietnam; nhacld.di11@vnu.edu.vn; 2 Academy of Journalism and Communication, Hanoi 123105, Vietnam; 3 Posts and Telecommunications Institute of Technology in Hanoi (PTIT), Hanoi 151100, Vietnam; nhannd@ptit.edu.vn; 4 Information Technology Institute, Vietnam National University in Hanoi (VNU-ITI), Hanoi 123105, Vietnam

**Keywords:** vehicle mode, driving event, smartphone sensor, motorbike assistance, optimized window size, optimized overlapping ratio

## Abstract

In this paper, we present a flexible combined system, namely the Vehicle mode-driving Activity Detection System (VADS), that is capable of detecting either the current vehicle mode or the current driving activity of travelers. Our proposed system is designed to be lightweight in computation and very fast in response to the changes of travelers’ vehicle modes or driving events. The vehicle mode detection module is responsible for recognizing both motorized vehicles, such as cars, buses, and motorbikes, and non-motorized ones, for instance, walking, and bikes. It relies only on accelerometer data in order to minimize the energy consumption of smartphones. By contrast, the driving activity detection module uses the data collected from the accelerometer, gyroscope, and magnetometer of a smartphone to detect various driving activities, i.e., stopping, going straight, turning left, and turning right. Furthermore, we propose a method to compute the optimized data window size and the optimized overlapping ratio for each vehicle mode and each driving event from the training datasets. The experimental results show that this strategy significantly increases the overall prediction accuracy. Additionally, numerous experiments are carried out to compare the impact of different feature sets (time domain features, frequency domain features, Hjorth features) as well as the impact of various classification algorithms (Random Forest, Naïve Bayes, Decision tree J48, K Nearest Neighbor, Support Vector Machine) contributing to the prediction accuracy. Our system achieves an average accuracy of 98.33% in detecting the vehicle modes and an average accuracy of 98.95% in recognizing the driving events of motorcyclists when using the Random Forest classifier and a feature set containing time domain features, frequency domain features, and Hjorth features. Moreover, on a public dataset of HTC company in New Taipei, Taiwan, our framework obtains the overall accuracy of 97.33% that is considerably higher than that of the state-of the art.

## 1. Introduction

Today, driving assistance and road safety are critical issues in all countries around the world. According to the global status report on road safety 2015 by the World Health Organization (WHO), road accidents are in the worldwide top-ten causes of death, killing more than 1.2 million of people per year. The road traffic fatality rates are especially high in the low-income and middle-income countries [1]. In fact, there are numerous factors possibly causing road accidents. Nonetheless, assisting and providing safety awareness for drivers during their trips is an effective approach to prevent such accidents.

Recently, researchers have paid a lot of attention to study various methods of providing assistance and safety awareness to drivers. Indeed, such works primarily fall into the following categories: recognizing vehicle mode (car, bus, train, bike, walking ...) [2,3,4,5,6,7,8,9], identifying driving styles (normal, aggressive, drunken, fatigue, drowsy, inattentive ...) [10,11,12,13,14,15,16], detecting normal/abnormal driving events (moving, stopping, turning left, turning right, weaving, sudden braking, fast u-turn...) [17,18,19,20,21,22], accident detection [23,24], estimating energy consumption and pollution [25], monitoring road and traffic condition [26,27,28,29,30].

In fact, there are several approaches to access driver and vehicle information. In the first approach, a set of sensors and additional hardware are pre-deployed in vehicles, for instance telematic boxes (e.g., black boxes provided by car insurance companies), on-board diagnosis (OBD-II) adapters plugged into the vehicle’s controller area network (CAN) [24,31]. The information recorded by these devices can be then retrieved or sent over the Internet. However, this strategy requires vehicles to install extra devices, which incur more cost. Moreover, it is not feasible to implement these techniques in certain types of vehicles like bikes, and motorbikes. To overcome these drawbacks, an alternative approach is to use smartphones to collect data through a set of embedded sensors such as inertial sensors (accelerometers and gyroscopes), global positioning systems (GPS), magnetometers, microphones, image sensors (cameras), light sensors, proximity sensors, direction sensors (compass) ... The technological advances and the rapid growth in smartphone usage make the latter approach become broadly used in recent studies.

Furthermore, the global status reports on road safety 2015 by WHO also shows that approximately a quarter of all road traffic deaths involve in motorcyclists. However, very few existing works provide driving assistance and safety awareness for motorcyclists [4,20,21,22,32]. Nonetheless, there are certain limitations in such works. The method proposed in [32] is constrained under certain conditions such as fixing the position of smartphones, and using some predefined threshold to distinguish between normal and abnormal driving patterns. However, such thresholds may be sensitive due to the variety of sensor quality in different smartphone models or road conditions. The work proposed in [4] must rely on the combination of GPS and accelerometer data to predict eight travelling modes. Nonetheless, its prediction accuracy is quite low with the average precision of 76.38% and an average recall of 75.88%.

In this work, we tend to develop a real-time flexible combined system, namely Vehicle mode-driving Activity Detection System (VADS), that is capable of detecting not only the vehicle mode currently used by a traveler (i.e., walking, a bike, a motorbike, a car, or a bus) but also various basic driving activities of travelers (i.e., stopping, going straight, turning left, turning right). In fact, the strategy of combining these two separating modules in our system allows improving the accuracy in recognizing driving events when the current vehicle mode of a traveler is known. The vehicle mode detection module simply relies only on accelerometer data in order to minimize the energy consumption of smartphones. However, the driving activity detection module accounts for turning left and turning right activities, which involve in changing the direction of vehicles. Hence, it requires the data from accelerometers, gyroscopes, and magnetometers. This work focuses on finding a solution that is able to collect sensor data and provide real-time prediction results in a smartphone application. Our system is thus designed to meet several main goals: it needs low computational resources and low energy consumption, and it needs to able to respond fast to the changes of travelers’ vehicle modes and driving activities.

It is well known that the data segmentation technique has been applied for activity recognition in which sensor data is split into a number of overlapping data segments (alternatively called data windows) of a predefined size. In fact, most of existing studies use the same window size and the same overlapping ratio for predicting all vehicle modes and all driving activities. Such parameter values are randomly chosen or taken from previous studies. Indeed, each vehicle mode as well as each driving activity has its own cyclic characteristics. Hence, it is unrealistic to fix such parameter values for all vehicle modes or all driving activities. Up to now, there exist few works that consider inferring the optimal data window size and the optimal overlapping ratio from training datasets [8,33,34]. The authors of [33,34] prove that the window size of 1–2 s results in the best accuracy and the best processing speed in predicting various human activities. Then, the authors of [8] show that the window size of 60 s leads to the highest overall recall rate in detecting vehicle modes from their dataset. However, this long window size causes a slow responding speed as well as a long processing time due to its long feature vectors. Thus, it is not suitable to apply this framework for real-time application. In this work, we thus propose an alternative algorithm to compute the optimal window size and the optimal overlapping ratio for each vehicle mode and each driving event from the training datasets. The obtained optimal window sizes fall in the range 4–6 s that are reasonable for real-time prediction. The inferred optimal parameters allow the vehicle mode detection module to improve its prediction accuracy by 2.73%, 3.04%, 6.45%, 7.37%, and 5.72% when using Random Forest, J48, Naïve Bayes, KNN, and SVM classifier, respectively, on a feature set combining time domain features, frequency domain features, and Hjorth features as comparing with the strategy using the same window size of 5 s and the same overlapping ratio of 50%. The similar improvements are observed in predicting various driving activities of motorcyclists.

The rest of the paper is organized as follows: the related work is summarized in Section 2. In Section 3, the detailed framework of our proposed VADS is described. Then, Section 4 provides the description of data processing and feature extraction processes carried out in this system. Next, we present the experimental settings and the evaluation of the system’s performance in detecting vehicle mode and driving activities in Section 5 and Section 6, respectively. Section 7 describes the performance comparison between our proposed framework and several recent works on a public dataset. Finally, we provide the conclusion remarks in Section 8.

## 2. Related Work

In this section, we review a number of recent works providing smartphone-based solutions for vehicle mode detection and driving event detection.

### 2.1. Vehicle Mode Detection

Table 1 provides the summary of existing works in the area of vehicle mode detection. The predicted vehicle modes, ranging from 3 to 8 modes, consist of the non-motorized ones (stationary, walk, run, bike) and the motorized ones (motorcycle, bus, car, train, tram, subway, ferry). Most of these works requires data from GPS or the combination of accelerometer with other sensors (gyroscope, magnetometer) [2,4,5,6,8]. Yet, these input requirements lead to higher power consumption of smartphones. Though there exist few works relying only on accelerometer data, their obtained prediction accuracy is low [3]. Numerous machine learning classification algorithms are applied, for instance, Random Forest (RF), Decision Tree (DT), Naïve Bayes (NB), K Nearest Neighbor (KNN), Support Vector Machine (SVM), Hidden Markov Models (HMM), Gradient boosting decision tree, and XGBoost. Among them, Random Forest usually results in the highest prediction accuracy [2,8]. The similar trend is observed in our experiments. In addition, several works achieving high prediction accuracy require a long window size. For example, the window size of 10 min and 60 s are used in the works of [5] and [8], respectively. As aforementioned, those prediction frameworks are not applicable for real-time prediction due to their long responding time and their high processing resource requirement.

### 2.2. Driving Event Detection

Table 2 presents the summary of recent studies in the area of driving event detection. It can be seen that all of these works require data from not only accelerometer but also other sensors. In fact, some driving events, such as left/right turns, involve changing the direction of vehicles. Such information cannot be obtained from only accelerometer data. In addition, the works allowing free position of smartphones usually need to convert input data from a phone’s coordinates into a vehicle’s coordinates [35,36,37]. Such requirement ensures the input data taken from multiple three-axis sensors are consistent. Along with numerous machine-learning algorithms (RF, SVM, Neural Network (NN), Artificial Neural Network (ANN), Bayesian Network (BN)), other methods like Dynamic Time Warping (DTW), fuzzy logic, and threshold detection are explored. Moreover, Random Forest is again shown to have the best prediction accuracy as comparing with other machine learning algorithms in detecting driving events [38]. Nonetheless, most of existing works target on only finding a driving event detection framework for car or bus drivers. Indeed, it is infeasible to extend some existing methods to identify driving events of motorcyclists, for instance, the methods using threshold detection techniques [35,36]. Though the method of Yu et al. [37] obtains high prediction accuracy, i.e., 96.88%, it requires too many features, i.e., 152 features. This factor certainly induces high computational time and resource. Thus, our work aims to investigate an efficient framework that can either provide real-time prediction with low computational resource or achieve high accuracy in detecting driving events of motorcyclists.

For more works on vehicle mode and driving event detection, we refer readers to the survey of Prelipcean et al. [9] and Engelbrecht et al. [39].

## 3. The Proposed Framework of Vehicle Mode-Driving Activity Detection System

**Figure 1:**
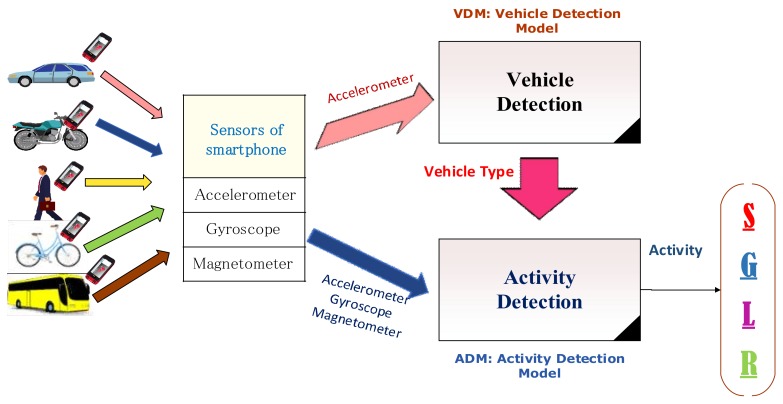
The Vehicle mode-driving Activities Detection System (VADS).

Our proposed system, VADS, consists of two main modules: The first one, Vehicle mode Detection Module (VDM), focuses on detecting the vehicle mode currently used by a user (i.e., walking, a bike, a motorbike, a car, or a bus) solely relying on accelerometer sensor data. The second one, Activity Detection Module (ADM), concentrates on detecting a set of primitive driving activities based on the data collected from accelerometer, gyroscope, and magnetometer sensors of smartphones when the user ‘s vehicle mode is known (Figure 1). This set contains the following activities: {stopping (S), going straight (G), turning left (L), turning right (R)}.

### 3.1. The Vehicle Mode Detection Module (VDM)

**Figure 2:**
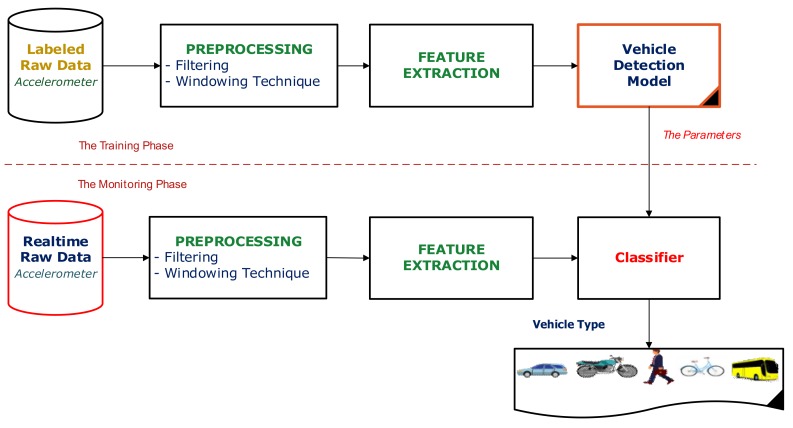
The framework of the Vehicle Detection Module (VDM).

In the details, VDM is divided into two phases, including the training phase and the monitoring phase (Figure 2). In the training phase, time series data are first collected from the accelerometer sensor and manually labeled with the corresponding vehicle type, i.e., walking, a bike, a bus, a car, or a motorbike. Then, several preprocessing techniques such as noise filtering and windowing technique are applied to calibrate the acceleration data. Next, representative information is extracted by exploring various categories of popular features, for example time domain features, and frequency domain features. A set of formulas for computing such features is presented in Section 4.2. The resulting feature vectors are then used to train the vehicle detection model. Finally, a number of popular machine learning classifying algorithms, such as Naïve Bayes, J48, Random Forest, SVM, and KNN are tested on the training dataset to select the most suitable classifier for the monitoring phase.

In the Monitoring phase, the real-time accelerometer data is captured, preprocessed, and then extracted into a set of relevant features as describing in the training phase. Finally, the type of vehicle currently used by a traveler is identified based on the best vehicle detection model built in the above training phase and the computed feature vectors.

### 3.2. The Activity Detection Module (ADM)

**Figure 3:**
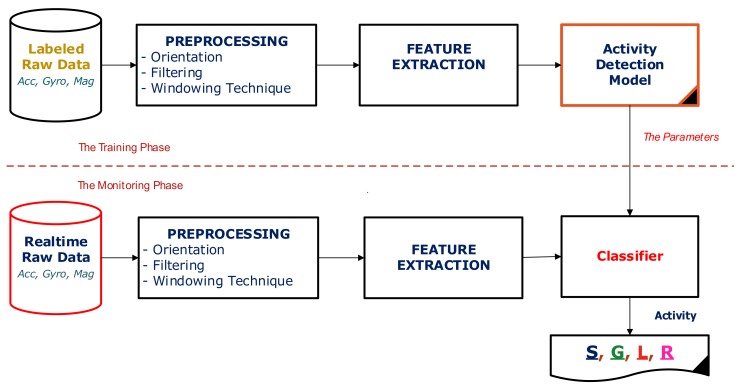
The framework of the Activity Detection Module (ADM).

As described above, ADM focuses on recognizing a set of basic driving activities for each vehicle mode, i.e., {Stopping, Going straight, turning Left, turning Right}. The structure of ADM is quite similar to VDM with two phases—the training phase and the monitoring phase (Figure 3).

Indeed, turning left and turning right activities involve the directional changes of vehicles. Thus, in order to adequately capture necessary information, ADM collects input data from the accelerometer, gyroscope, and magnetometer. In addition, there are several changes in the Preprocessing and Feature extraction processes in the framework of ADM. In the preprocessing process, the raw accelerometer data is reoriented from a smartphone’s coordinates into the vehicle’s coordinates in order to accurately receive the information about the directional changes of vehicles. In the feature extraction process, a number of additional features representing the angle changing information of vehicle are introduced.

## 4. Materials and Methods

### 4.1. Data Preprocessing

#### 4.1.1. Data Filtering

Filtering is performed to mitigate noisy data values before the feature extraction process that is important to enable better recognition. There are different types of filters that can be applied to noisy sensor data. In general, a low-pass filter is usually used to remove some high frequency noise interfering with the inertial sensor data. The transfer function of a first order low-pass filter in discrete domain can be represented by

(1) $$H(z)=1+\alpha z^{-1},$$

where *α* is a factor that determines the cutoff frequency of the filter and *z*^−1^ represents for the unit delay between data samples [40]. Higher order filters can be used to build a low-pass filter or high-pass filter or band-pass filter with better filtering characteristics. However, the computational complexity increases with the order of filter. Therefore, in order to minimize this problem, a second-order filter with following transfer function is often used [40], as follows:

(2) $$H(z)=\frac{Y(z)}{X(z)}=\frac{b_{0}+b_{1}z^{-1}+b_{2}z^{-2}}{1+a_{1}z^{-1}+a_{2}z^{-2}},$$

where *a_i_* and *b_i_* are the filter coefficients that determine type of the filter. In this article, we use a band-pass filter based on the second-order filter to select a given frequency band which is significant to increase the importance of some features like Hjorth parameters. In particular, the coefficients [*b*_0_, *b*_1_, *b*_2_] are [1, 0, ±1] and [*a*_1_, *a*_2_] are [−1.56, 0.6].

#### 4.1.2. Reorientation

As briefly aforementioned, the orientation of smartphones might change during a trip, and this change becomes a challenge in activity recognition. Thus, to solve this problem and obtain consistent accelerometer data for the later activity recognition process, two approaches can be used.

The first approach is to use the orientation-independent features that are often based on the magnitude of the acceleration calculated as follows:

(3) $$a_{mag}=\sqrt{a_{x}^{2}+a_{y}^{2}+a_{z}^{2}},$$

where *a_x_*, *a_y_*, *a_z_* are the acceleration components along axes. This solution is simple and cost-effective due to using the acceleration alone in recognition, which means that the solution can be applied for inexpensive smartphones where only an accelerometer is installed.

**Figure 4:**
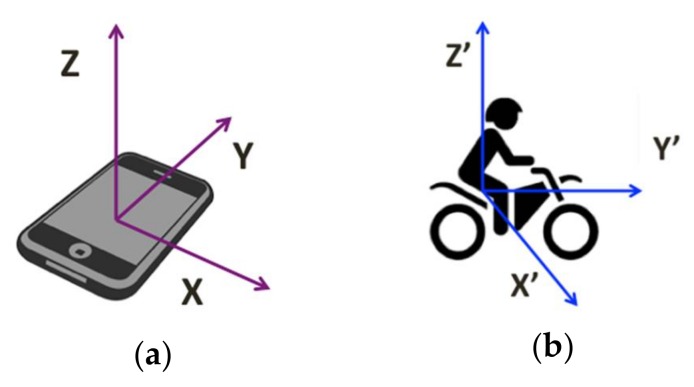
(**a**) The orientation of a smartphone given by (*X*, *Y*, *Z*) coordinate system. (**b**) The orientation of a vehicle given by (*X*’, *Y*’, *Z*’) coordinate system.

The second approach is required to transform the collected accelerometer data, representing the acceleration measurement in m/s^2^ along *X*, *Y*, *Z* axes (Figure 4a), from a smartphone’s coordinate system to a vehicle’s coordinate system, representing by *X*’, *Y*’, *Z*’ axes (Figure 4b), through angular rotations around three axes, as below.

The vehicle’s coordinates can be obtained from the accelerometer data collected on the smartphone’s coordinates by the following formula [41]:

(4) $$\left(\begin{array}{c} a_{X^{\prime }}\\ a_{Y^{\prime }}\\ a_{Z^{\prime }} \end{array}\right)=R\left(\begin{array}{c} a_{X}\\ a_{Y}\\ a_{Z} \end{array}\right)$$

where **R** = **R_x_** × **R_y_** × **R_z_** and **R_x_**, **R_y_**, **R_z_** are the rotation matrices representing the rotation of the sensor data around the corresponding axes. The corresponding rotation matrices are given by

(5) $$R_{x}=\left(\begin{array}{ccc} 1 & 0 & 0\\ 0 & \cos\beta & -\sin\beta \\ 0 & \sin\beta & \cos\beta \end{array}\right)$$

(6) $$R_{y}=\left(\begin{array}{ccc} \cos\alpha & 0 & -\sin\alpha \\ 0 & 1 & 0\\ \sin\alpha & 0 & \cos\alpha \end{array}\right)$$

(7) $$R_{z}=\left(\begin{array}{ccc} \cos\varphi & \sin\varphi & 0\\ -\sin\varphi & \cos\varphi & 0\\ 0 & 0 & 1 \end{array}\right)$$

where *β*, *α*, and *φ* are respectively the rotation angles around three axes *X*’, *Y*’, and *Z*’, which can be calculated and updated by retrieving the gravity and magnetic data. In smartphones, the gravity sensor is a virtual sensor that is derived from the accelerometer with the help of gyroscope and magnetometer [41]. Although the reorientation allows the accelerometer signal to be collected at any orientation of the smartphone, the cost of this solution is higher than that of the first solution due to requiring smartphones with fully equipped inertial sensors.

Our work focuses on the investigation of both solutions and shows the importance of reorientation in activity classification module. Indeed, as these approaches do not use GPS signal, they thus minimizes the battery consumption needed.

#### 4.1.3. Sliding Window

**Figure 5:**
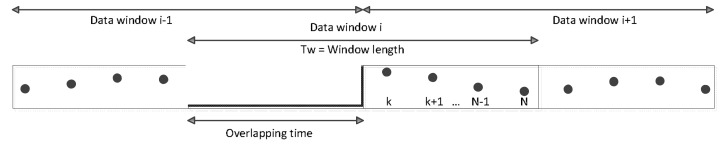
Each data window consists of N signal values. Two consecutive data windows overlap each other by an overlapping ratio of 50%.

As described above, after applying noise filtering and/or reorienting processes, the resulting acceleration data is split into a number of smaller data segments of a predefined size. Each data segment is alternatively called a data window. Two consecutive data windows overlap each other by a certain amount of signal values (Figure 5). Indeed, an overlapping ratio is commonly used to present such an overlapping segment.

Most of the existing studies follow the static sliding window approach, which uses a fixed window size for different vehicle mode detection as well as different activity recognition. However, each vehicle mode has its own cyclic variation of data patterns. In fact, the duration of sliding windows influences on the prediction accuracy. If the sliding window size is too small, some periodic characteristics can be lost; the system performance of classifiers is thus degraded. By contrast, if the window size is too large, the noise interference from other vehicle modes can be increased. Therefore, it is necessary to investigate the effect of sliding window size and overlapping ratio on the classification process.

Given a vehicle mode, a classifier, a feature set, an overlapping ratio of data windows, and a threshold (denoted Δ_t_), the procedure of inferring the optimized sliding window size from the training dataset is demonstrated in Algorithm 1. The area under the ROC curve (AUC) generated by measuring the sensitivity versus specificity is used as the metric for the optimization process. The Algorithm 1 works as the followings: first, it initializes the window size to 1 s—the minimum window size used in many existing studies. Then, the window size is iteratively increased by a number of seconds (denoted *v*), if it results in a prediction accuracy improvement being higher or equal to the threshold Δ_t_ in terms of the AUC measure. Otherwise, the incremental loop stops. The finally obtained window size value is the optimal one.

Note that the threshold Δ*_t_* indicates the minimum prediction accuracy improvement considered as a significant change, and *v* is the incremental value of the window size in each iterative loop. Thus, they do not depend on the characteristics of vehicle modes and driving activities. In our experiments, Δ*_t_* and *v* are respectively set to 0.001 and 1. The function *ComputeAUC(w)* is responsible for calculating the accuracy of predicting a given vehicle mode/driving activity in term of the AUC measure for the window size *w*, a given classifier, a given feature set, a given window overlapping ratio.

**Algorithm 1:** ComputeOptimalWindowSize(Δ_t_,v)
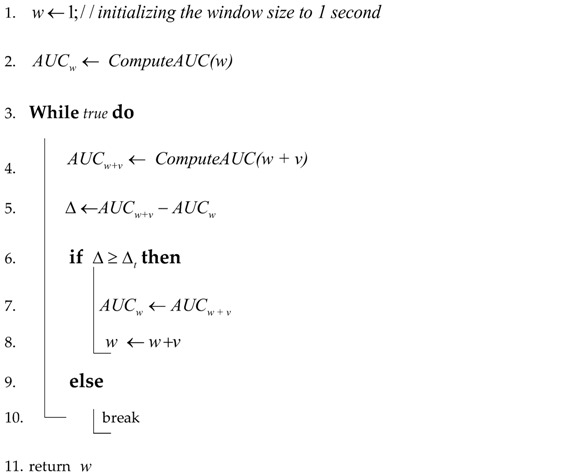


In this work, the above algorithm is run with the different overlapping ratios of 75%, 50%, and 25%. The combination of the window size and the overlapping ratio resulting in the highest AUC is chosen as the optimized parameters.

### 4.2. Features Extraction

Feature selection affects significantly to the performance of activity recognition classifier. The features including time-domain metrics and frequency-domain metrics are extracted in our work to perform the classification algorithm efficiently.

In the time domain, the popular features are based on statistic metrics such as mean, variance, and standard deviation. Besides, some other features in time domain like difference between the minimum and maximum values, zero-crossings, cross-correlation, peak to average ratio (PAR), signal magnitude area (SMA), signal vector magnitude (SVM), and differential signal vector magnitude (DSVM) are additionally computed.

The PAR metric is calculated by

(8) $$PAR=\frac{\max\left(a\right)}{\mu }$$

where *a* is the applied component and *μ* is the mean of corresponding component in each window. The correlation between two data streams can be calculated as follows:

(9) $$R=\frac{\sum_{k=1}^{N}\left(a_{i}^{k}-\mu_{i}\right)\left(a_{j}^{k}-\mu_{j}\right)}{\sqrt{\sum_{k=1}^{N}{\left(a_{i}^{k}-\mu_{i}\right)}^{2}\sum_{i=1}^{N}{\left(a_{j}^{k}-\mu_{j}\right)}^{2}}}$$

where *i*, *j* = *x*, *y*, *z* and *i* ≠ *j*, *N* is the number of data values in each window. The SMA, SVM, and DSVM are computed as the summation of time integrals of accelerometer components respectively according to the following formulas:

(10) $$SMA=\frac{1}{2T}\sum_{k=2}^{N}\left(\left|a_{i}^{k-1}\right|+\left|a_{i}^{k}\right|\right)\times \left(t_{k}-t_{k-1}\right)$$

(11) $$SVM=\frac{1}{N}\sum_{k=1}^{N}\sqrt{a_{k}^{2}}$$

(12) $$DSVM=\frac{1}{2T}\sum_{k=2}^{N}\left(\left|a_{k-1}^{\prime }\right|+\left|a_{k}^{\prime }\right|\right)\times \left(t_{k}-t_{k-1}\right)$$

where $a_{k}^{\prime }=a_{k+1}-a_{k}$, *t* is the time instance, and *T* is the duration of a data window.

In the frequency domain, the time series data of each component is converted by using the Fast Fourier Transform (FFT) algorithm. Then, the features consisting of energy and entropy metrics are computed. The spectral energy in certain frequencies is an additional feature computed as follows:

(13) $$E_{FFT}=\sum_{k=n1}^{n2}{\left|X(k)\right|}^{2}$$

where *X* is the FFT of the accelerometer data and *n*_1_ and *n*_2_ are the indices of FFT coefficients in certain frequencies. In our work, the spectral energy at 1 to 3 Hz is extracted to characterize the vehicle mode. The entropy metric is computed by

(14) $$H=-\sum_{k=1}^{N}p_{k}\log_{2}\left(p_{k}\right)\;\mathrm{with}\;p_{k}=\frac{\left|X[k]\right|}{\sum_{k=1}^{N}\left|X[k]\right|}$$

This feature is significant in differentiation between the statuses with similar energy.

Hjorth parameters consisting of activity (*A*), mobility (*M*), and complexity (*C*) were introduced by Hjorth in 1970 to analyze time series data [42]. Because these parameters can provide useful information in both the time and frequency domains, they were mostly used in analyzing the biomedical signals such as ECG, EEG. Therefore, these parameters are proposed as additional features in our work. The activity that provides the power information is computed by following formula:

(15) $$A=\frac{\sum_{k=1}^{N-1}d_{0k}^{2}}{N-1}$$

where $d_{0k}=a_{k+1}-a_{k}$. The mobility which provides an estimate of the mean frequency is given as

(16) $$M=\sqrt{\frac{m_{1}}{A}}$$

where $m_{1}=\sum_{k=1}^{N-2}d_{1k}^{2}/\left(N-2\right)$ and $d_{1k}=d_{0,k+1}-d_{0,k}$. The complexity which provides an estimate of the bandwidth is given as

(17) $$C=\sqrt{\frac{m_{2}}{m_{1}}}$$

where $m_{2}=\sum_{k=1}^{N-3}d_{2k}^{2}/\left(N-3\right)$ and $d_{2k}=d_{1,k+1}-d_{1,k}$. Applying these formulas on the acceleration time series, we obtain the corresponding features.

For each data window, a set of features from the raw or transformed accelerometer data is calculated. Beside three components of accelerometer along *x*, *y*, *z* axes, an addition component is computed as

(18) $$a_{rms}=a_{mag}-g,$$

where *a_mag_* is computed by (3) and *g* is the gravity calculated by averaging the magnitude of the accelerometer data over several time windows. This component is equivalent to the rms value of linear accelerometer data. Table 3 lists all features and the applied components used in this study.

In the VDM, for each data window, we compute sets of features in every domain that consists of a set of 20 features (T1), a set of four features (F1), and a set of only three Hjorth features of rms component (H1). T1 consists of mean, variance and standard deviation along three axes (nine features), difference and zero-crossings along three axes (six features), cross-correlation coefficient along three axes (three features), signal vector magnitude and mean of the rms component. F1 contains energy features along three axes and an rms component. Then we also create combined sets of features from different domains: a combined set of 24 features (TF1), a combined set of 23 features (TH1), and a set of 27 features (TFH1).

In the ADM, the activity is more complex that requires the ability to detect the change of orientation in modes of turning left and turning right. We propose to additionally use physical components that are the orientation angles calculated from tri-axis accelerometer data for extracting new features. The rotation angles according to the change in orientation for the k^th^ signal in a data window can be approximately estimated as the following [43]:

(19) $$\varphi [k]=\tan^{-1}\left(\frac{a_{y}^{k}}{\sqrt{{\left(a_{x}^{k}\right)}^{2}+{\left(a_{z}^{k}\right)}^{2}}}\right)$$

(20) $$\theta [k]=\tan^{-1}\left(\frac{-a_{x}^{k}}{a_{z}^{k}}\right)$$

Then, newly added features can be computed from these components by applying the metrics such as mean, variance and integration. Thus, the features like PAR, SMA, and DSVM are also added to obtain a set T2 of 34 features in the time domain. Furthermore, the entropy features are also added to obtain a new set F2 of 07 features and TF2 of 41 features consisting of 24 features of TF1 and 17 new features. Set H2 additionally includes 18 features of Hjorth parameters. We also build combined sets of 59 features for activity recognition. All sets of features are summarized in Table 4.

## 5. Vehicle Mode Recognition

### 5.1. Data Collection

An android-based program was developed to collect data from the tri-axis accelerometer sensor. We collected five datasets on 10 volunteer subjects in the age of 22–40 years old with different vehicle modes. Each dataset contains the samples for only one of five vehicle modes. The subjects freely put their smartphone at any place while travelling, for example in their shirt pocket, trouser pocket, handbag, holding in hand or in their vehicle’s hold. In the meantime, they were asked to tag the corresponding vehicle mode as walking, a bicycle, a motorbike, a car, or a bus. Table 5 shows the data sets collected for each vehicle mode. The sensor signal was recorded such that we obtained 50 samples per second. The raw accelerometer data were then transformed from the phone’s coordinates to the vehicle’s coordinates according to the method described in Section 4.1.2. Both the raw and transformed accelerometer data are recorded in two separate files for comparison.

Different types of smartphones are used to demonstrate the validity and reliability of our framework in activity recognition. In order to assess the generalization capability of our framework, the datasets were collected in different conditions similar to those that occur in reality. In each vehicle mode, the samples were collected during the trip in urban area when the subjects used different vehicles at various speeds in both bad and good road conditions.

### 5.2. Experiment Designs

First, we investigate the effect of feature sets and classification algorithms in vehicle mode detection. The raw accelerometer data are split into a number of windows of 5 s with 50% overlapping. For each window, a vector of features, previously mentioned in Section 4.2, were extracted. We use six feature sets—T1, F1, H1, TF1, TH1, TFH1—as described in Table 4 for comparison to evaluate the performance of our VDM.

Once the datasets were prepared, we used the WEKA tool to predict the vehicle mode. In each case, the default setting was used. Because there are different classification algorithms which is best suited for specific recognition problems, we investigated five classifiers—i.e., Random Forest (RF), Naïve Bayes (NB), Decision tree J48, K Nearest Neighbor (KNN), Vector Support Machine (SVM)—to select the best algorithm for VDM. For evaluating the accuracy (performance) of each classification algorithm, we used 10-fold cross validation and the accuracy and the area under the curve (AUC) as the accuracy metrics of our model. The accuracy of classification is the proportion of correctly classified examples of a specific class out of all its examples [44], whereas AUC is generated by measuring the sensitivity versus specificity [45].

(21) $$Accuracy\;=\;\frac{TP+TN}{TP+FP+TN+FN}$$

where *TP*, *TN*, *FP*, and *FN* respectively represent the number of true positive, true negative, false positive, and false negative samples.

### 5.3. Results and Discussion

#### 5.3.1. Effect of Feature Sets on Vehicle Mode Detection

**Figure 6:**
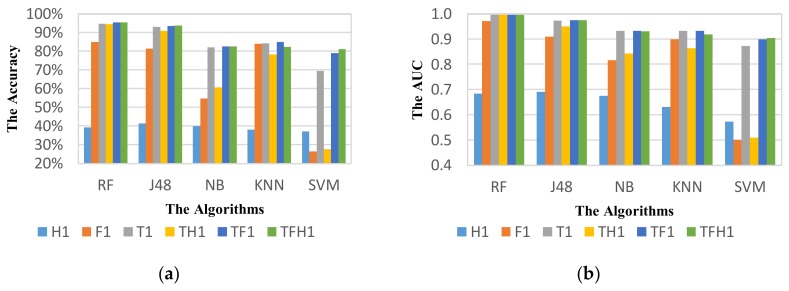
The performance of VDM on different classifiers, and different feature sets with the window size of 5 s at 50% overlapping based on the metric: (**a**) Accuracy; (**b**) AUC.

Figure 6 shows the recognition performance of VMD module on classifiers using different feature sets. In general, the random forest algorithm is the best classifier with most of feature sets in terms of accuracy and AUC. For the feature set H1 with three Hjorth parameters only, the J48 is the best classifier and the followings are NB, RF KNN, and SVM. The specific accuracy values of classifiers on different feature sets are shown in Table 6. The results show that the features in time domain contribute most to the performance. Because there are only three features in the set H1, the recognition accuracy of all classifiers is relatively low. While the accuracy of classifiers RF and J48 using the feature set of time domain T1 can attain higher than 90%. In general, the addition of other features to the set can improve the performance of VDM in most of classifiers.

In particular, the performance of VMD attained an accuracy of 94.76% for the RF algorithm using the set T1. A small improvement in accuracy and AUC can be obtained by using the feature sets TF1 and TFH1 combined with Hjorth parameters. The same trends can be observed for the J48 NB and the SVM algorithms in terms of accuracy. However, the performance of VDM in terms of AUC attains the best result using the set TF1, although the difference is very small.

However, the KNN algorithm achieves the best result on the feature set TF1 in terms of both accuracy and AUC. The addition of Hjorth parameters to the feature set TFH1 does not improve the performance. Indeed, it degrades the performance of detection. This result is attributed to the correlation of Hjorth parameters to other features in time and frequency domains, which causes the misleading detections.

#### 5.3.2. Parameter Optimization in Vehicle Mode Detection

**Figure 7:**
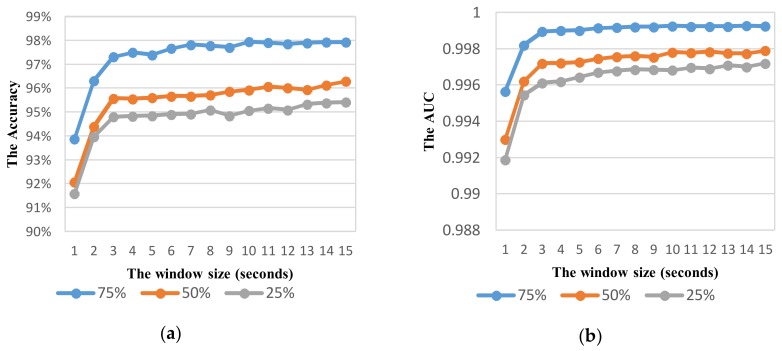
Effect of window size on the vehicle mode detection system performance with different amount of overlapping based on the metric: (**a**) Accuracy; (**b**) AUC.

Next, we investigate the effect of the sliding window size and overlapping ratio on the performance. The RF classifier using the feature set TFH1 is used in this investigation due to its best performance obtained in previous investigation. Figure 7 shows the performance of VDM as a function of the window size at three overlapping ratios of 75%, 50%, and 25%. In terms of both accuracy and AUC metrics, the increase in window size improves the performance of detection and the overall/average accuracy of recognition trends to be saturated at the window size of larger than 6 s. When the longer window size is, the more information of vehicle mode is captured for recognition.

Generally, the overlapping ratio of 75% outperforms all other overlapping ratios at all sliding window sizes. At windows of longer 6 s, the average accuracy of higher 97.7% is obtained for 75% overlap. While the highest accuracy for 50% and 25% overlaps are 96.29% and 95.42% respectively. A good result of 75% overlap can be attributed by reduction of sensitivity on transitions between moving status in each vehicle mode.

**Figure 8:**
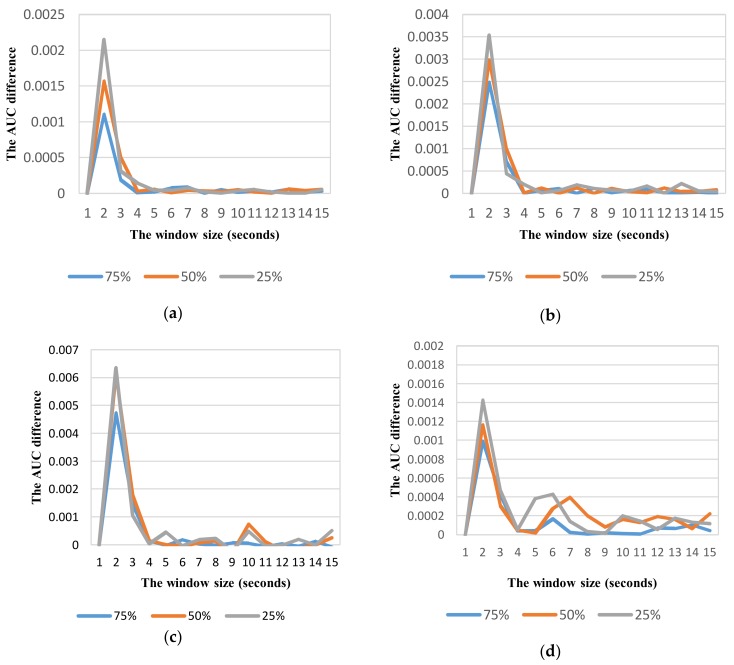
Variation of AUC difference between two consecutive window sizes at different overlapping ratios for different vehicle detection: (**a**) Car; (**b**) Motorbike; (**c**) Bus; (**d**) Walking (Non-vehicle).

By analyzing the performance difference in terms of AUC between two consecutive window sizes, the relevant window size for each vehicle mode can be determined. Figure 8 shows the change of performance between two consecutive window sizes in each vehicle mode at different overlaps. In all vehicle modes, the change is relatively small (less than 0.001) at the windows longer than 4 s. Therefore we used a range of 6 s that is sufficient to observe about two windows of each vehicle mode for selecting optimum parameters in each class. Table 7 shows the optimum parameters of sliding window for each class of vehicle mode based on the best AUC metric. The window of 6 s with 75% overlap is selected for all vehicles except in car mode. Intuitively, the optimum window is shorter when the average speed of vehicle is higher. However, the optimum windows of motorbike, bicycle, and bus modes are similar due to the similarity in movement patterns of these vehicle modes in urban roads without dedicated lanes. By contrast, the car often travels in dedicated lanes that result in a movement at faster speed.

After determining optimized parameters, the performance of VDM is reevaluated with selected parameters as shown in Table 7. The optimized set of parameters improves the average recognition accuracy up to 98.33% for RF classifier. The summary results for predicting vehicle mode presented as the confusion matrix are shown in Table 8 to indicate the accuracy in each detection class. Specially, the accuracy for detecting car mode can go up to ~100%. Though the accuracy for detecting bike mode is lowest, it remains high with about 96.87% cases being correct. The accuracy for detecting motorbike, bus and walking modes are respectively 97.95%, 96.97%, and 97.08%. The reason for lower accuracy in detecting bike, motorbike and bus modes can be explained as follows: when the motorbike subject had to slow down in crowded or bad roads, the accelerometer patterns between driving and walking are very similar, hence a number of driving status were misclassified as walking. A smaller number of bike and motorbike modes were also misclassified as walking mode when the motorbike subject used the automatic-gear motorbike at a constant slow speed. As mentioned above, the similarity of moving patterns in different vehicle modes is very challenging for detection in real-life conditions. We note that only raw accelerometer data is used in this investigation. However, high performance of vehicle detection is obtained.

**Figure 9:**
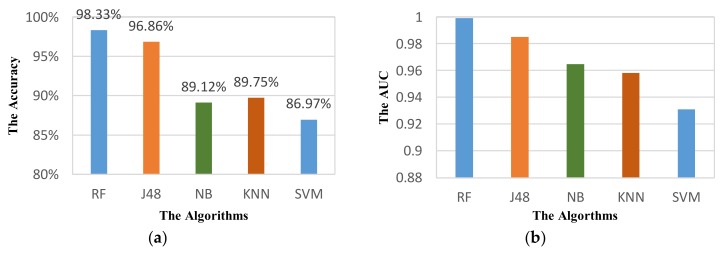
Performance of VDM with the optimized parameters: (**a**) Accuracy; (**b**) AUC.

We also used these parameters for other classifiers to demonstrate the effectiveness of our selection. Figure 9 shows the performance of VDM on different classifiers. The results show a considerable improvement on performance of recognition for all classifiers. In terms of accuracy, the performance of VDM can increase by 2.73%, 3.04%, 6.45%, 7.37%, and 5.72% for RF, J48, NB, KNN, and SVM respectively. In terms of AUC metric, the highest increase in performance is nearly 0.04 for KNN, while it is about 0.01 for J48. The higher recognition performance of the system means the smaller increase in performance.

## 6. Activity Mode Recognition

### 6.1. Data Collection and Experiments

In activity mode recognition, we have collected the data on motorbike as an example of our proposal. The motorbike mode is a challenging mode in cities, especially in developing countries due to its freedom style of moving. By using the same android-based collection program, we collected four datasets from three volunteer subjects in the age of 22–40 years old with different activity modes (Table 9). In our study, we consider four basic activities of travelling mode: Stop (ST), Going Straight (GS), Turning Left (TL), and Turning Right (TR). Each collected dataset contains the samples for only one of four activity modes in motorbike moving scheme. Because the activity modes such as TL and TR often take place at lower speeds, duration of a data example for these activities does not exceed about 5 to 6 s that is different from ST and GS modes. Although the subjects’ smartphones can be located in different positions, they are only hold in the subjects’ hands to handle the sensor data recording in ST, TL and TR modes.

First, we investigate the effect of feature sets and classification algorithms in activity mode detection. Because the activity detection is more complicated than the vehicle mode detection, we additionally use more feature sets beside the sets used in VDM. In this investigation, the raw accelerometer data are split into a number of data windows of 5 s with 50% overlapping ratio. Then, the effect of sliding window is investigated to select relevant parameters for ADM. The data is also processed in the same way as the data in VDM.

### 6.2. Results and Discussion

#### 6.2.1. Effect of Feature Sets and Classification Algorithms on Activity Mode Detection

**Figure 10:**
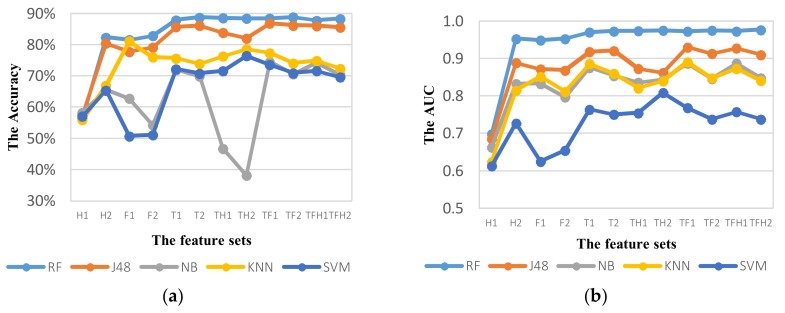
The activity mode detection system performance of classifiers using different feature sets with the window size of 5 s at 50% overlapping based on the metric: (**a**) Accuracy; (**b**) AUC.

Firstly, we compare the performance of ADM using different feature sets in different domains. Figure 10 shows the performance of ADM using different feature sets with the window size of 5 s at 50% overlapping. Table 10 summarizes all obtained results from different classifiers with various feature sets. In general, RF algorithm outperforms all the other classifiers over all feature sets.

For RF classifier, the addition of new features improves the performance of ADM in all domains. In set H2 with addition of Hjorth parameters in three axis components and angular components, the performance is considerably improved by 25.93% compared to using the set H1. The accuracy of ADM using only Hjorth features can go up to 82.39% that demonstrates the importance of orientation components to extract features in predicting TL and TR modes. Similarly, ADM based on sets T2, TF2 and TFH2 performs better than sets T1, TF1, and TFH1, respectively. Although the accuracy of ADM based on TFH2 is higher than that of ADM based on TFH1, the accuracy with additional Hjorth features is slightly less that shows a correlation between Hjorth features and other features in feature sets. However, the best performance of ADM with 50% overlap is obtained by the set TFH2 in terms of AUC or by the set TF2 in terms of accuracy.

The tendency of accuracy for J48 classifier is similar to RF classifier, but the result shows a decrease in AUC when new features are added. The best performance for J48 classifier is obtained by the set TF1 in terms of both AUC and accuracy. For the NB, KNN, and SVM algorithms, the addition of new features influences more obviously to the decrease in performance of ADM. On the other words, the NB, KNN, and SVM algorithms are much more sensitive to the correlation of features. This influence is resulted from the features extracted in the same components in the same domain (for example, the rotation angle features are computed from the same three-axis accelerometers components). Therefore, ADM using the set TF1 provides the best performance for NB, KNN, and SVM classifiers. However, their accuracy does not exceed 80%. Because RF algorithm selects a random subset of features in each branch, it can reduce sensitivity of classifier to correlated features.

**Figure 11:**
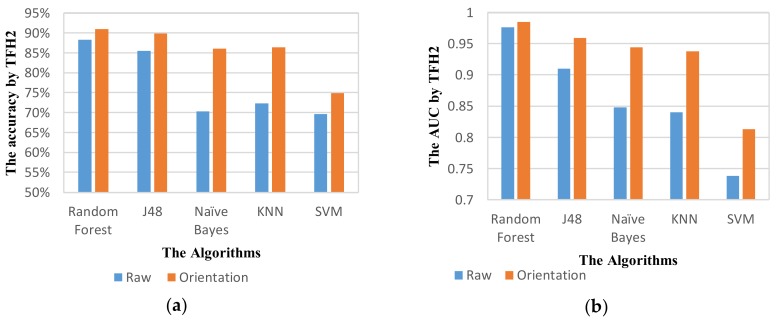
The activity mode detection system performance of different classifiers using raw and transformed data with the window size of 5 s at 50% overlapping on TFH2 feature set based on the metric: (**a**) Accuracy; (**b**) AUC.

Orientation of smartphone can be significant in detection of movement direction. Hence, we investigate the performance of ADM using raw data and transformed data with the same parameters. Figure 11 and Table 11 show the performance results of ADM using the feature set TFH2. The results show an improved performance of ADM using transformed accelerometer data in all classifiers. For RF classifier, the accuracy of recognition goes up to higher than 90%. The NB and KNN classifiers show a high increase by ~15.7% and ~14% respectively in performance using transformed data. With these results, we can demonstrate the importance of reorientation in recognizing complex activities such TL and TR modes.

#### 6.2.2. Parameter Optimization in Activity Mode Detection

**Figure 12:**
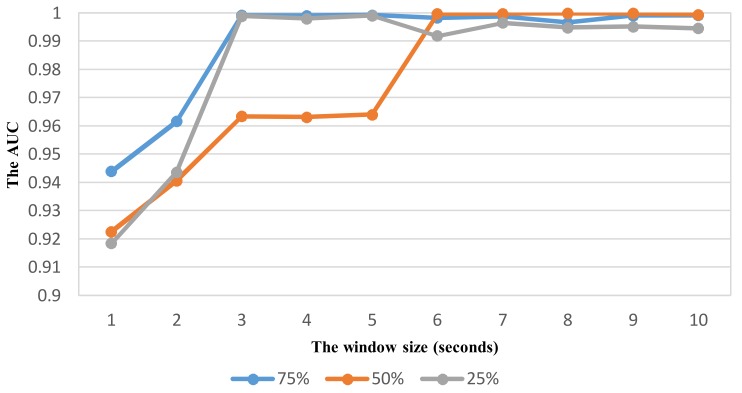
The performance result (AUC) of detecting the activity Stopping with respect to window size and overlapping ratio.

**Figure 13:**
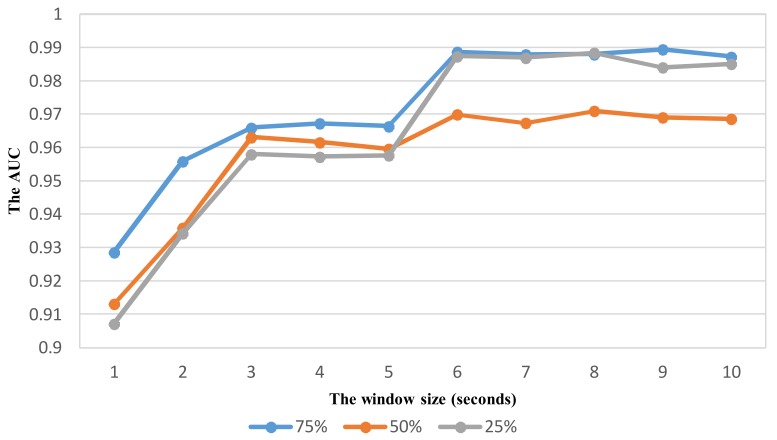
The performance result (AUC) of detecting the activity Going with respect to window size and overlapping ratio.

**Figure 14:**
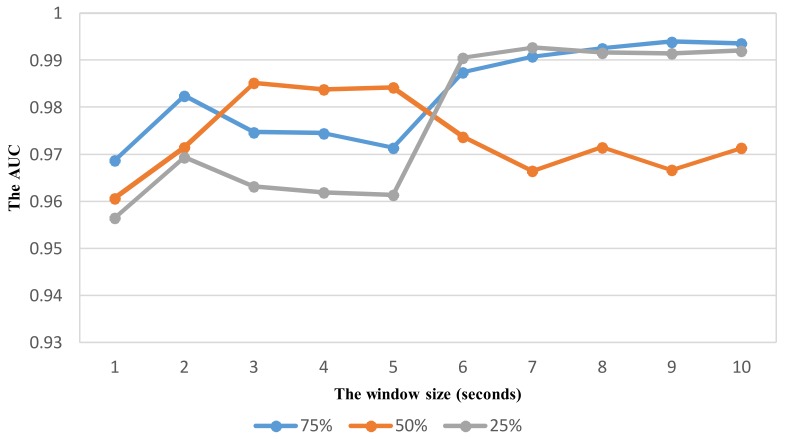
The performance result (AUC) of detecting the activity Turning left with respect to window size and overlapping ratio.

**Figure 15:**
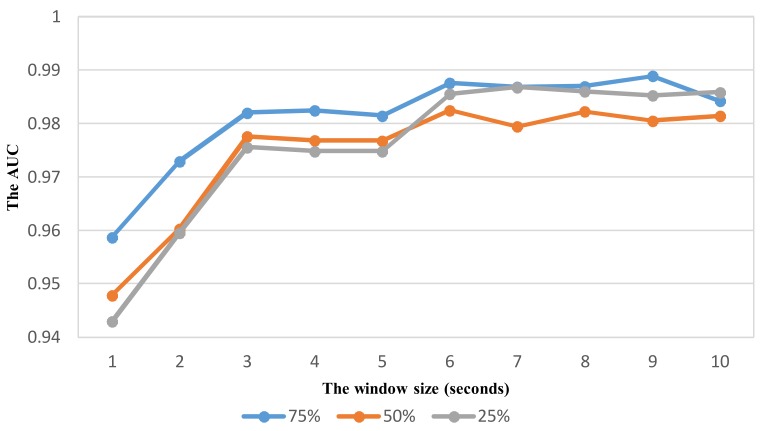
The performance result (AUC) of detecting the activity Turning right with respect to window size and overlapping ratio.

**Figure 16:**
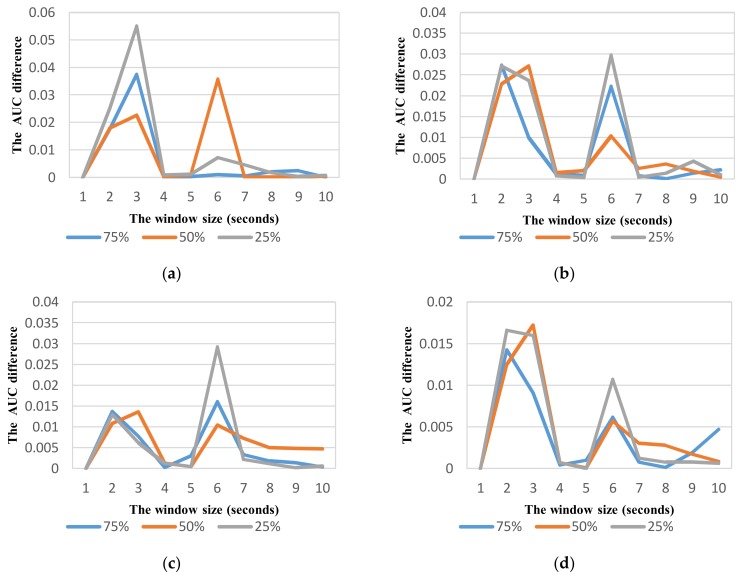
Variation of AUC difference between two consecutive window sizes at different overlapping ratios for activity mode detection: (**a**) Stop; (**b**) Going straight; (**c**) turning Left; (**d**) turning Right.

As shown in vehicle mode detection, the parameter optimization is very important to improve the recognition performance. Using the same way similar to VDM, the effect of sliding window with different overlaps on the performance is investigated to select relevant parameters for ADM using the feature set TFH2. The recognition performance with the widow size in the range from 1 to 10 s is shown in Figure 12, Figure 13, Figure 14 and Figure 15 for each corresponding activity mode. Similar to the case of VDM, there is little change of the performance result in each activity mode when the window size is longer than 6 s. On the other words, the performance difference in terms of AUC between two consecutive window sizes is negligible at windows longer than 6 s as shown in Figure 16. Hence, the optimum parameters are selected by investigating the performance in terms of AUC in the range of window size from 1 to 6 s. The selected parameters for individual activity mode are shown in Table 12 The obtained results show the optimum overlap ratio for ST and GS modes is 75% that is different from that of 50% for TL and TR modes. The window size of 4 s is optimum for ST mode, while longer window size is required for other activity modes.

**Figure 17:**
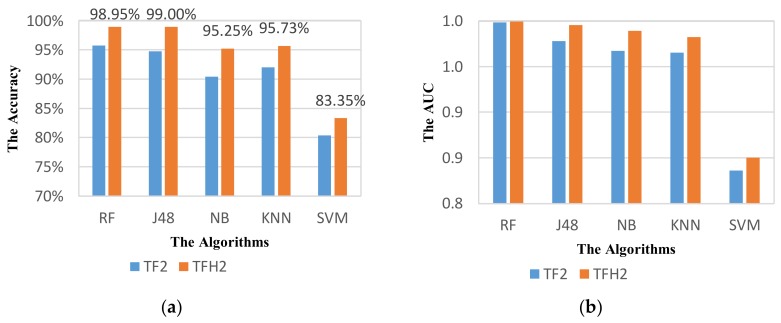
Performance of activity mode detection system with the optimized parameters using different classifiers based on the metric: (**a**) Accuracy; (**b**) AUC.

Based on the selected parameters, the performance of ADM is reevaluated. These parameters are applied for not only RF classifier but also other classifiers. The obtained results are shown in Figure 17. Figure 17 shows an obvious improvement of the performance in all classifiers using the feature sets TF2 and TFH2. For RF classifier, the highest accuracy of 98.95% is obtained that is increased by 7.98%. For other classifiers except SVM, the accuracy can reach higher 95%. These results also demonstrate the importance of optimizing parameters in activity mode recognition. It is interesting that the performance of ADM using the set TFH2 with optimum parameters is much higher than that using the set TH2 which is quite different in case of non-optimized recognition.

## 7. Performance Comparison with the Recent Works

In this section, we provide a comparison between our proposed framework and several existing works on a dataset recently collected by HTC company [46]. Up to date, this is the only publicly available dataset which consists of various transportation modes (still, walk, run, bike, bus, car, metro, train, tram, HSR). Yet, there exist only a few works validating their proposed methods on this dataset [6,8,46]. Nonetheless, the authors of [47] concentrate on differentiating between non-motorized modes (still, walk, run, and bike) and being on a vehicle. The two remaining frameworks detect either non-motorized modes (still, walk) or motorized modes (bus, car, metro, train, tram, and HSR) relying on data collected from accelerometer, magnetometer, and gyroscope. In fact, it has been proved that among these three sensors, accelerometer consumes the lowest amount of power [46]. Thereafter, our method, based on only accelerometer data, certainly requires less power than those of [6,8]. As previously mentioned, their frameworks require a long window size, i.e., 17.06 s and 60 s, that leads to a longer responding time and a higher computational resource as comparing with our framework. Moreover, the approach proposed in [8] must rely on a very large feature set containing 348 features. It is thus infeasible to be implemented in real-time prediction application. Note that our vehicle mode detection module requires only 27 features. In addition, Table 13 shows that on the dataset of HTC company, our method achieves the overall prediction accuracy of 97.33% which is significantly higher than the best method of two recent works. In the meantime, our model requires less computational time as comparing with the one proposed in [6].

## 8. Conclusions

In this work, we propose a flexible combined system that is composed of two modules: one to detect the vehicle mode of users, one to detect the instant driving events regardless the orientation and the position of smartphones. Our system achieves the average accuracy of 98.33% in detecting the vehicle modes, and the average accuracy of 98.95% in recognizing the driving events of motorcyclists when using the Random Forest classifier, and a feature set consisting of time domain features, frequency features and Hjorth features. Moreover, the experimental results indicate that the optimal parameters (window size and overlapping ratio) lead to a considerable increment of the system performance as comparing to the approach using the same window size of 5 s and the overlapping ratio of 50%. In detail, the vehicle mode detection module improves its prediction accuracy by 2.73%, 3.04%, 6.45%, 7.37%, and 5.72% when respectively using Random Forest, J48, Naïve Bayes, KNN, and SVM classifiers. Similarly, the activity detection module gains the prediction accuracy by 7.98%, 9.06%, 8.60%, 9.33%, and 8.48% for respectively Random Forest, J48, Naïve Bayes, KNN, and SVM classifiers. Note that the optimal window sizes inferred by Algorithm 1 range from 4 to 6 s, which are feasible for real-time application. Furthermore, Naïve Bayes, KNN, and SVM classifiers are shown to be quite sensitive to the correlation of features as the driving event prediction accuracy decreases when more features are added. By contrast, Random Forest and J48 classifiers do not suffer from such effects.

## Figures and Tables

**Table 1:** Summary of recent researches on vehicle mode detection.

Studies	Modes	Smartphone Data	Algorithm	Features	Window Size	Prediction Accuracy
Bedogni et al. [2]	walk, car, train	gyroscope, accelerometer	RF, SVM, NB	time-domain	10 s	Accuracy: 97.71%
Hemminki et al. [3]	stationary, bus, train, metro, tram, car	accelerometer	HMM	time-domain, frequency-domain, peak-based, segmented-based	1.2 s	Precision: 84.9% Recall: 85.3%
Widhalm et al. [4]	bus, car, bike, tram, train, subway, walk, motorcycle	gps, accelerometer	HMM	time-domain, frequency-domain	≤2 min	Precision: 76.38% Recall: 75.88%
Shafique and Hato [5]	walk, bike, bus, car, train, subway	gyroscope, accelerometer	RF	time-domain	10 min	Accuracy: 99.96%
Fang et al. [6]	high speed rail (HSR), metro, bus, car, train	accelerometer, magnetometer, gyroscope	KNN, DT, SVM	time-domain	17.06 s	Accuracy: 83.57%
Xiao et al. [7]	walk, bike, bus, car, train, subway	gps	KNN, DT, SVM, RF, Gradient boosting decision tree, XGboost	global/local	-	Accuracy: 90.77%
Guvensan et al. [8]	stationary, walk, bus, car, tram, metro, train, ferry	accelerometer, magnetometer, gyroscope	RF, KNN, J48, NB, Healing	time-domain	60 s	Precision: 94.95% Recall: 91.63%

**Table 2:** Summary of recent researches on driving event detection.

Studies	Driving Events	Smartphone Data	Methods	Features	Coordinate Reorientation	Prediction Accuracy
Johnson and Trivedi [11]	normal/abnormal driving events (left/right turn, u-turn, left/right swerving)	accelerometer, gyroscope, magnetometer, gps, video	DTW	x,y,z-acceleration, gyroscope, Euler angle rotation	smartphones are fixed	TP: 91%
Castignani et al. [15]	hard acceleration, hard braking, over speeding, aggressive steering	accelerometer, magnetometer, gravity, gps	fuzzy logic	the time derivative (jerk) of the acceleration magnitude, speed variation, bearing variation, the average yaw rate, the jerk standard deviation	Yes	TP > 90%
Ma et al. [35]	speeding, irregular driving direction change, abnormal speed control	accelerometer, gyroscope, gps, microphone	threshold detection	computing speed from gps and y-acceleration, detecting direction change based on z-gyroscope, and turn signal based on audio signal	Yes	Precision: 93.95% Recall: 90.54%
Li et al. [36]	abnormal speed changing, steering, weaving, operating smartphone during driving	accelerometer, gyroscope	threshold detection	yaw angle	Yes	TP > 90%
Yu et al. [37]	weaving, swerving, sideslipping, fast u-turn, turning with a wide radius, sudden braking	accelerometer, orientation sensor	SVM, NN	152 time-domain features	Yes	Accuracy: 96.88%
Júnior et al. [38]	aggressive braking, aggressive acceleration, aggressive left/right turn, aggressive left/right lane changing, non-aggressive events	accelerometer, magnetometer, gyroscope, linear acceleration	ANN, SVM, RF, BN	Time-domain: mean, median, standard deviation, increase/decrease tendency	smartphones are fixed	AUC: 0.980–0.999

TP—True Positive, AUC—Area Under the Curve.

**Table 3:** Feature lists used in the proposed VADS.

Type	Features	Definition	Applied Components
Statistic	*μ*	Mean	*a_x_*, *a_y_*, *a_z_*, *a_rms_*, *φ*, *θ*
Time domain	*σ* ^2^	Variance	*a_x_*, *a_y_*, *a_z_*, *φ*, *θ*
	*σ*	Standard deviation	*a_x_*, *a_y_*, *a_z_*
	Diff = max(*x*) − min(*x*)	Difference	*a_x_*, *a_y_*, *a_z_*
	R	Cross correlation	*(a_x_*, *a_y_)*, *(a_x_*, *a_z_)*, *(a_z_*, *a_y_)*
	ZC	Zero crossings	*a_x_*, *a_y_*, *a_z_*
	PAR	Peak to average ratio	*a_x_*, *a_y_*, *a_z_*
	SMA	Signal magnitude area	*a_x_*, *a_y_*, *a_z_*, *a_rms_*
	SVM	Signal vector magnitude	*a_rms_*
	DSVM	Differential signal vector magnitude	*a_rms_*
	I	Integration	*φ*, *θ*
Hjorth parameters	A	Activity	*a_x_*, *a_y_*, *a_z_*, *a_rms_*, *φ*, *θ*
	M	Mobility	*a_x_*, *a_y_*, *a_z_*, *a_rms_*, *φ*, *θ*
	C	Complexity	*a_x_*, *a_y_*, *a_z_*, *a_rms_*, *φ*, *θ*
Frequency domain	E_FFT_	Energy	*a_x_*, *a_y_*, *a_z_*, *a_rms_*
	En	Entropy	*a_x_*, *a_y_*, *a_z_*

**Table 4:** Sets of features.

Domains	Set of Features	Number of Features	Applied Module
Time (T)	T1	20	VDM
Frequency (F)	F1	04	VDM
Hjorth (H)	H1	03	VDM
T + F	TF1	24	VDM
T + H	TH1	23	VDM
T + F + H	TFH1	27	VDM
Time	T2	34	ADM
Frequency	F2	07	ADM
Hjorth	H2	18	ADM
T + F	TF2	41	ADM
T + H	TH2	52	ADM
T + F + H	TFH2	59	ADM

**Table 5:** Datasets for VDM.

Vehicle Mode	Number of Subjects	Total Recording Time	Positions of Smartphone
Car	3	400 min	In hand, in pockets, in box
Bike	2	300 min	In hand, in pockets
Motorbike	3	500 min	In hand, in pockets, in bag
Bus	4	500 min	In hand, in pockets, in bag
Walking	4	200 min	In hand, in pockets

**Table 6:** The performance of VDM in terms of accuracy on different classifiers and different feature sets with the window size of 5 s at 50% overlapping.

	RF	J48	NB	KNN	SVM
**H1**	39.41%	41.43%	40.07%	38.03%	37.13%
**F1**	85.04%	81.36%	54.66%	84.09%	26.54%
**T1**	94.76%	93.08%	82.09%	84.33%	69.64%
**TF1**	95.47%	93.65%	82.56%	85.01%	78.97%
**TH1**	94.64%	91.10%	60.64%	78.39%	27.73%
**TFH1**	95.60%	93.82%	82.67%	82.38%	81.25%

**Table 7:** Optimized parameters of VDM.

	Car	Bike	Motorbike	Walking	Bus
**Window size (seconds)**	5	6	6	6	6
**Overlapping ratio**	75%	75%	75%	75%	75%
**AUC**	0.9998646	0.9989851	0.9990746	0.9994278	0.9984576

**Table 8:** Confusion matrix with the optimized parameters.

a	b	c	d	e	Class
5334	0	0	0	0	a = Car
1	1979	24	15	24	b = Bike
2	17	2924	10	32	c = Motorbike
2	11	9	1700	29	d = Walking
0	13	22	24	1890	e = Bus

**Table 9:** Datasets for ADM.

Activity Mode	Number of Subjects	Total Recording Time	Positions of Smartphone
Stopping (ST)	3	10 min	In hand
Going Straight (GS)	2	20 min	In hand, in pockets
Turning Left (TL)	3	30 min	In hand
Turning Right (TR)	3	10 min	In hand

**Table 10:** The performance of ADM using different feature sets with the window size of 5 s at 50% overlapping.

	Random Forest		J48		Naïve Bayes		KNN		SVM	
	ACC	AUC	ACC	AUC	ACC	AUC	ACC	AUC	ACC	AUC
H1	56.46%	0.6986	58.33%	0.6854	58.33%	0.6635	56.04%	0.6243	57.07%	0.6126
H2	82.39%	0.9531	65.57%	0.8881	65.57%	0.8326	66.89%	0.8153	65.41%	0.7273
F1	81.60%	0.9490	77.84%	0.8720	62.80%	0.8330	81.13%	0.8520	50.79%	0.625
F2	82.85%	0.9530	79.16%	0.8690	54.29%	0.7970	75.99%	0.8120	51.12%	0.6551
T1	87.93%	0.9706	71.99%	0.9182	71.99%	0.8756	75.69%	0.8860	72.28%	0.7643
T2	88.79%	0.9730	69.90%	0.9213	69.90%	0.8546	73.91%	0.8596	70.86%	0.7506
TH1	87.80%	0.974	83.84%	0.8730	46.77%	0.8360	76.32%	0.8210	71.64%	0.7550
TH2	88.39%	0.975	82.06%	0.8620	38.19%	0.8440	78.56%	0.8400	76.45%	0.8090
TF1	88.39%	0.9727	74.52%	0.9303	74.52%	0.8869	77.34%	0.8914	73.60%	0.7683
TF2	88.85%	0.9752	70.60%	0.9134	70.60%	0.8462	74.08%	0.8481	70.99%	0.7384
TFH1	87.80%	0.9733	74.25%	0.9278	74.25%	0.8874	74.87%	0.8738	71.62%	0.7577
TFH2	88.32%	0.9768	70.36%	0.9104	70.36%	0.8479	72.39%	0.8406	69.64%	0.7384

**Table 11:** The result between Raw data and Orientated data using TFH2 feature set with the window size of 5 s at 50% overlapping.

	Random Forest		J48		Naïve Bayes		KNN		SVM	
	AUC	ACC	AUC	ACC	AUC	ACC	AUC	ACC	AUC	ACC
**Raw data**	0.97676483	88.32%	0.910449	85.55%	0.847943	70.36%	0.840576	72.39%	0.738373	69.64%
**Orientation data**	0.9854096	90.97%	0.959214	89.94%	0.944504	86.05%	0.937778	86.40%	0.813128	74.87%

**Table 12:** The optimized parameters of ADM.

	S	G	L	R
**Window size (seconds)**	4	6	5	6
**Overlapping**	75%	75%	50%	50%
**AUC**	0.99942150231399	0.992827782904118	0.996841188906852	0.987251082251082

**Table 13:** The prediction accuracy of the proposed method and the previous studies on the dataset of HTC company [46]. The notion “-” indicates that data is not provided.

	Overall Prediction Accuracy	Computational Cost (μs)	Model Size (KB)
Fang et al. [6] (using KNN)	83.57%	9550	106,300
Guvensan et al. [8] (using RF)	91.63%	-	-
Our proposed method (using RF)	97.33%	4.9	187
